# Virion Structure of Israeli Acute Bee Paralysis Virus

**DOI:** 10.1128/JVI.00854-16

**Published:** 2016-08-26

**Authors:** Edukondalu Mullapudi, Antonín Přidal, Lenka Pálková, Joachim R. de Miranda, Pavel Plevka

**Affiliations:** a Structural Virology, Central European Institute of Technology, Masaryk University, Brno, Czech Republic; b Department of Zoology, Fishery, Hydrobiology, and Apidology, Faculty of Agronomy, Mendel University in Brno, Brno, Czech Republic; c Department of Ecology, Swedish University of Agricultural Sciences, Uppsala, Uppsala, Sweden; University of Maryland

## Abstract

The pollination services provided by the western honeybee (Apis mellifera) are critical for agricultural production and the diversity of wild flowering plants. However, honeybees suffer from environmental pollution, habitat loss, and pathogens, including viruses that can cause fatal diseases. Israeli acute bee paralysis virus (IAPV), from the family Dicistroviridae, has been shown to cause colony collapse disorder in the United States. Here, we present the IAPV virion structure determined to a resolution of 4.0 Å and the structure of a pentamer of capsid protein protomers at a resolution of 2.7 Å. IAPV has major capsid proteins VP1 and VP3 with noncanonical jellyroll β-barrel folds composed of only seven instead of eight β-strands, as is the rule for proteins of other viruses with the same fold. The maturation of dicistroviruses is connected to the cleavage of precursor capsid protein VP0 into subunits VP3 and VP4. We show that a putative catalytic site formed by the residues Asp-Asp-Phe of VP1 is optimally positioned to perform the cleavage. Furthermore, unlike many picornaviruses, IAPV does not contain a hydrophobic pocket in capsid protein VP1 that could be targeted by capsid-binding antiviral compounds.

**IMPORTANCE** Honeybee pollination is required for agricultural production and to sustain the biodiversity of wild flora. However, honeybee populations in Europe and North America are under pressure from pathogens, including viruses that cause colony losses. Viruses from the family Dicistroviridae can cause honeybee infections that are lethal, not only to individual honeybees, but to whole colonies. Here, we present the virion structure of an Aparavirus, Israeli acute bee paralysis virus (IAPV), a member of a complex of closely related viruses that are distributed worldwide. IAPV exhibits unique structural features not observed in other picorna-like viruses. Capsid protein VP1 of IAPV does not contain a hydrophobic pocket, implying that capsid-binding antiviral compounds that can prevent the replication of vertebrate picornaviruses may be ineffective against honeybee virus infections.

## INTRODUCTION

The agricultural production of most flowering food crops depends on the pollination services provided by the western honeybee (Apis mellifera) (1). Furthermore, honeybee pollination is also critical for maintaining the ecological and genetic diversity of wild plants (2). However, winter honeybee colony mortality has been increasing in North America and Europe over the last 2 decades, leading to a decline in the number of honeybee colonies that is becoming a serious threat to the adequate provision of pollination services and food security (3–5). Honeybees suffer from habitat loss, intensified agricultural management, pesticides, parasites, and pathogens, including numerous viruses that contribute to the collapse of honeybee colonies (6).

The viruses that have the greatest impact on honeybee populations are small icosahedral picorna-like viruses from the families Dicistroviridae and Iflaviridae (7). Israeli acute paralysis virus (IAPV) is an Aparavirus from the family Dicistroviridae. IAPV, Kashmir bee virus (KBV), and acute bee paralysis virus (ABPV) constitute a group of closely related viruses that are distributed worldwide, with different members predominating in different geographical regions (8). Infections by IAPV and related viruses decrease the longevity of individual bees and endanger the survival of whole colonies. Furthermore, IAPV infection decreases the homing ability of foraging honeybees, which are not able to find their way back to the hive (9). The spread of the viruses is accelerated by transmission by a parasitic mite, Varroa destructor (7, 8, 10). IAPV has been linked with colony collapse disorder in the United States (11), while ABPV has been associated with similar rapid adult bee depopulation phenomena in Europe (8, 12).

Viruses from the family Dicistroviridae have nonenveloped icosahedral virions containing a linear, single-stranded, positive-sense RNA genome 8,500 to 10,200 nucleotides in length (13). The genome of dicistroviruses includes two nonoverlapping open reading frames (ORFs), ORF1 and ORF2, which encode polyproteins containing nonstructural and structural (capsid-forming) proteins, respectively. The polyproteins are cotranslationally and posttranslationally cleaved by viral proteases to produce functional subunits. The capsid proteins originating from a single polyprotein precursor form a protomer—the basic building block of the capsid. Previously, structures of two dicistroviruses from the genus Cripavirus, triatoma virus (TrV) and cricket paralysis virus (CrPV), were determined (14–17). Protomers, as well as icosahedral asymmetric units of dicistroviruses, consist of subunits VP1 to -4. The major capsid proteins VP1 to -3 form the capsid shell, with pseudo-T=3 icosahedral symmetry, whereas VP4 is a small protein attached to the inner surface of the capsid. The major capsid proteins have the jellyroll β-sandwich fold common to many other virus capsid proteins. Dicistrovirus virions assemble as immature particles containing the precursor protein VP0, which is presumed to be cleaved into VP4 and VP3 after the particles are filled with the RNA genome, similar to the situation in picornaviruses (18, 19).

In order to initiate infection, virus genomes need to be released from virions and transferred across the biological membrane into the cell cytoplasm. There is limited information about this process in viruses from the family Dicistroviridae. However, related enteroviruses from the family Picornaviridae have been extensively studied as model organisms for genome delivery (20–25). The genome release of enteroviruses is preceded by structural changes of the capsid, leading to the formation of an expanded A particle that is induced by receptor binding or by the low pH of late endosomes (20, 26–28). The A particles contain pores at the icosahedral 2-fold symmetry axes (21–26, 29–31) that allow the release of the genome and VP4 subunits and the exposure of the N-terminal region of VP1 subunits on the virion surface (26, 32, 33).

Here, we present the crystal structures of the IAPV virion, the first structurally characterized representative of the genus Aparavirus. In addition, we determined the structure of the IAPV pentamer of the capsid protein protomers produced by capsid disassembly after genome release.

## MATERIALS AND METHODS

### Virus propagation in honeybee pupae.

The propagation of IAPV was carried out as described in the COLOSS BeeBook (34). Brood areas with A. mellifera white-eyed pupae were identified by the color and structural features of the cell caps. White-eyed pupae were carefully extracted from the brood combs so as not to injure the pupae. The pupae were placed on paper furrows with their ventral side up. In total, 2,262 pupae were used for IAPV propagation. The virus inoculum (1 μl) was injected into pupae with a Hamilton micropipette with a 30-gauge 22-mm-long needle through the intersegmental cuticle between the 4th and 5th sternites. Pupae that leaked hemolymph after the injection were discarded. The optimal concentration of the virus in the inoculum for virus production was determined experimentally by comparing virus yields when using different virus concentrations in the injection inoculum. Inoculated pupae were placed into petri dishes with paper furrows and incubated at 30°C and 75% humidity for 5 days. Typical IAPV-induced darkening was observed in 80% of the injected pupae (35). After incubation, the pupae were frozen at −20°C. For long-term storage, the pupae were kept at −80°C.

### Virus purification.

Fifty experimentally infected honeybee pupae were homogenized with a Dounce homogenizer in 30 ml of phosphate-buffered saline (PBS), pH 7.5 (Sigma-Aldrich). The nonionic detergent NP-40 was added to a final concentration of 0.5%, and the homogenate was incubated for 1 h at room temperature. The extract was centrifuged at 8,000 × *g* for 30 min. The pellet was discarded, and the supernatant was centrifuged at 150,000 × *g* for 3 h in a Ti50.2 fixed-angle rotor (Beckman-Coulter). The resulting pellet was resuspended in PBS to a final volume of 5 ml. MgCl_2_ was added to a final concentration of 5 mM, as well as 20 μg/ml DNase I and 20 μg/ml RNase. The solution was incubated at room temperature for 30 min and centrifuged at 4,000 × *g* for 15 min. The resulting supernatant was loaded onto a CsCl (0.6-g/ml) solution prepared in PBS. The ultracentrifugation proceeded for 16 h to establish the CsCl gradient. Virus bands were collected by gentle piercing of the ultracentrifuge tubes with an 18-gauge needle. The viruses were transferred to PBS by several rounds of concentration and dilution using centrifuge filter units with a 100-kDa molecular mass cutoff. This procedure yielded about 300 μg of virus with a purity sufficient for crystallization screening. Sample purity with respect to contaminating honeybee viruses was checked by reverse transcription-quantitative PCR (RT-qPCR), using previously reported virus-specific assays (34). In both preparations, the total sum of contaminating viruses was less than 1% of the virus of interest. The nucleotide sequences of the virus preparations were determined by sequencing 300 ng of RNA, purified using a Qiagen RNA purification kit, by IonTorrent (Thermo Fisher Scientific) technology and standard protocols for library preparation and sequencing. The IonTorrent reads were mapped to the IAPV GenBank reference sequence NC_009025 using Tmap v4.4.8, included in TorrentSuite 4.4.2, with Life Technologies-recommended parameters. Variability and consensus sequences were created using mpileup from samtools v.0.1.8 and an in-house script.

### IAPV crystallization.

IAPV crystallization screening was performed at 20°C using the virus dissolved in PBS at a concentration of 3 mg/ml. Approximately 1,800 crystallization conditions were tested by the sitting-drop vapor diffusion method in 96-well plates. Cuboid crystals with a longest dimension of approximately 0.05 mm were obtained in 0.1 M cadmium chloride, 0.1 M Na acetate, pH 4.5, and 15% (vol/vol) polyethylene glycol (PEG) 400. These crystals were flash frozen in liquid nitrogen without additional cryoprotectant and used to collect diffraction data. Crystals of a different type with a rhombic shape and a longest dimension of approximately 0.2 mm were obtained in 20% PEG 10,000, 8% ethylene glycol, 0.1 M HEPES, pH 7.5. These crystals diffracted X rays to a resolution of 2.7 Å. However, subsequent analysis revealed that they were composed of pentamers of IAPV capsid protein protomers.

### IAPV structure determination and refinement.

IAPV crystallization produced two types of crystals: (i) P2_1_2_1_2, containing one-half of a virus particle in the crystallographic asymmetric unit, and (ii) the P2_1_2_1_2_1_ crystal form, which did not contain a virus particle. Instead the P2_1_2_1_2_1_ crystallographic asymmetric unit contained two pentamers of capsid protein protomers, whose bases faced each other. The orientations of the virion and of the pentamers in the crystals were determined using the programs GLRF and Phaser (36, 37).

The P2_1_2_1_2 crystal form was solved initially. Self-rotation function plots and packing considerations indicated that 1/2 of a virus particle occupied a crystallographic asymmetric unit. The IAPV virion was positioned with one of the icosahedral 2-fold axes superimposed on the crystallographic 2-fold axis. The orientation of the virion was determined in a one-dimensional locked-rotation function search with the icosahedral symmetry in starting orientation, defined as described by Rossmann and Blow, rotated around the *y* coordinate axis (36, 38). Reflections at between 5.0- and 4.5-Å resolution were used for the calculations. The radius of integration was set to 140 Å. The results suggested that the virion is rotated (Φ = 90°, ϕ = 90°, and κ = 12.57°) from the standard icosahedral orientation according to the polar-angle convention. The position of the center of the particle was identified in a one-dimensional translation function search using the program Phaser (37). An appropriately oriented and positioned CrPV model (Protein Data Bank [PDB] code 1B35) was used to calculate phases up to a resolution of 10 Å using the program CNS (39). The phases were refined by 25 cycles of real-space electron density map averaging by the program AVE (40), using the 30-fold noncrystallographic symmetry. Phase extension was applied in order to obtain phases for higher-resolution reflections. Addition of a small fraction of higher-resolution data (one index at a time) was followed by three cycles of averaging. This procedure was repeated until phases were obtained for all the reflections to 4.0-Å resolution.

The electron density map corresponding to an icosahedral asymmetric unit from the P2_1_2_1_2 crystal (the virion) was used as a molecular replacement model for the phasing of the P2_1_2_1_2_1_ crystal form (the pentamers). Phase extension was applied in order to obtain phases for reflections in the 4- to 2.7-Å resolution range. The electron density of the capsid protein protomer was then used to phase the P2_1_2_1_2 (virion) crystal form.

The initial model, derived from the CrPV structure converted to polyalanine, was subjected to manual rebuilding using the programs Coot and O and to coordinate and B factor refinement using the program CNS (simulated annealing, gradient minimization, and individual B factor refinement) (39, 41, 42). Noncrystallographic symmetry constraints were enforced during the refinement. The model of the capsid proteins was built in the P2_1_2_1_2_1_ crystal form, using data to a resolution of 2.7 Å. The model could not be built for residues 1 to 58 and 259 to 318 of VP2, because the corresponding electron density was not resolved in the map. No density corresponding to VP4 could be identified in the P2_1_2_1_2_1_ crystal form.

The model building for the P2_1_2_1_2 crystal form that contained the IAPV virion was started from the model built in the P2_1_2_1_2_1_ crystal form. The main differences between the two crystal forms were in the structure of the N terminus of VP2 and in the electron density for IAPV VP4. The VP4 model was built as a polyalanine. The P2_1_2_1_2 model was refined using dynamic energy network (DEN) restraints to the pentamer structure of the P2_1_2_1_2_1_ crystal form. No water molecules were added to the P2_1_2_1_2 crystal model due to the limited resolution of the diffraction data. All the measured reflections were used in the refinement of the P2_1_2_1_2 crystal. If calculated, the *R*_free_ value would be very similar to the *R* value, due to the 30-fold noncrystallographic symmetry present in the diffraction data (43).

### Determination of the effect of IAPV proteins on liposome integrity.

Liposomes composed of phosphatidylcholine, phosphatidylethanolamine, lysophosphatidylcholine, sphingomyelin, phosphatidylserine, and phophatidylinositol (Avanti Polar Lipids) in molar ratios (43:23:13:9:6:6) filled with the self-quenching fluorescent dye carboxyfluorescein were prepared as described previously (44). The fluorescence quantum yield of the dye encapsulated in liposomes is about 5% of that obtained when the liposomes are disrupted and the dye is released and diluted in the medium. The percentage of dye release induced by addition of detergent or IAPV was determined by measuring fluorescence at an excitation wavelength of 485 nm and an emission wavelength of 520 nm, as described previously (44).

### Accession numbers.

The atomic coordinates of the IAPV virion, together with the structure factors and phases obtained by phase extension, were deposited in the Protein Data Bank under the code 5CDC. The IAPV pentamer was deposited as 5CDD. The consensus nucleotide sequences of the IAPV preparation were deposited in GenBank under accession number EF219380.

## RESULTS AND DISCUSSION

### Structures of the IAPV virion and capsid proteins.

**FIG 1:**
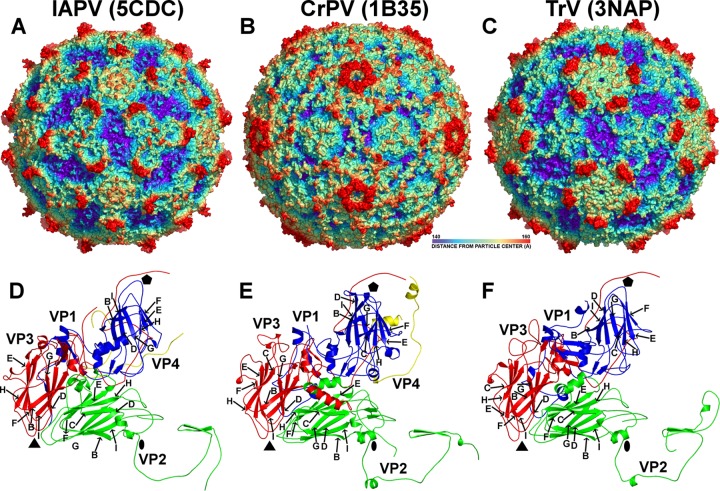
Comparison of virion and capsid protein structures of IAPV, CrPV, and TrV. (A to C) The molecular surfaces of IAPV (A), CrPV (B), and TrV (C) virions are colored based on the distance from the virion center. The depressions are shown in blue and protrusions in red. (D to F) Cartoon representations of the capsid protein protomers of IAPV (D), CrPV (E), and TrV (F). VP1 subunits are shown in blue, VP2 in green, VP3 in red, and VP4 in yellow. The names of β-strands of IAPV capsid proteins are shown. The positions of the 5-fold, 3-fold, and 2-fold icosahedral symmetry axes are indicated by pentagons, triangles, and ovals, respectively.

The crystal structures of the IAPV virion and of the pentamer of capsid protein protomers were determined to resolutions of 4.0 Å and 2.7 Å, respectively (Table 1). The maximum outer diameter of the IAPV virion is 340 Å (Fig. 1A). The IAPV capsid is built from major capsid proteins VP1, VP2, and VP3 arranged in a pseudo-T=3 icosahedral symmetry (Fig. 1D). VP1 subunits form pentamers around 5-fold axes, while VP2 and VP3 subunits constitute alternating heterohexamers around the icosahedral 3-fold axes (Fig. 1D). The three major capsid proteins have jellyroll β-sandwich folds with β-strands named, according to the picornavirus convention, B to I (Fig. 1D) (45). Two antiparallel β-sheets forming the cores of the subunits contain strands BIDG and CHEF, respectively. The N termini of the major capsid proteins are located inside the capsid, while the C termini are exposed on the virion surface. The minor capsid protein VP4 is attached to the inner face of the capsid (Fig. 1D). A complete model of the IAPV icosahedral asymmetric unit could be built, except for residues 1 to 5 of VP1, 1 to 15 and 259 to 318 of VP2, and 1 of VP3. The minor capsid protein VP4 has 69 residues; however, due to the lack of features in the corresponding regions of the electron density map, it was modeled as a 45-residue-long polyalanine chain.

**TABLE 1:** Crystallographic data collection and refinement statistics

Crystallization condition	IAPV pentamer^*a*^	IAPV virion^*b*^
Space group	P2_1_2_1_2_1_	P2_1_2_1_2
Wavelength (Å)	0.9998	0.9998
a, b, c (Å)	112.2, 274.2, 288.3	343.1, 383.3, 329.9
α, β, γ (°)	90, 90, 90	90, 90, 90
Resolution (Å)^*c*^	70–2.7 (2.79–2.70)	70–4.0 (4.07–4.00)
*R*_merge_^*c*^	0.086 (0.51)	0.21 (0.63)
<I>/<σI>^*c*^	13.2 (2.7)	4.7 (1.5)
Completeness (%)^*c*^	98.7 (99.5)	72.7 (54.8)
Redundancy	4.2	2.5
No. of reflections	240,313	250,379
*R*_work_/*R*_free_	24.4/25.1	30.6^*e*^
No. of atoms	5,617	6,116
RMSD^*d*^ bond length (Å)	0.013	0.015
RMSD bond angle (°)	1.49	1.42
Ramachandran favored (%)^*f*^	90.0	79.1
Ramachandran allowed (%)^*f*^	9.3	19.1
Ramachandran outliers (%)^*f*^	0.7	1.8
Poor rotamers (%)^*f*^	2.7	2.6
Cβ deviation (%)	0.9	0.1

a 0.1 M cadmium chloride, 0.1 M sodium acetate, pH 4.5, 15% (vol/vol) PEG 400.

b Twenty percent PEG 10,000, 8% ethylene glycol, 0.1 M HEPES, pH 7.5.

c Values in parentheses are for the highest resolution shell.

d RMSD, root mean square deviation.

e All reflections were used in the refinement. The *R*_free_, if it were calculated, would be very similar to *R*_work_ because of the 30-fold noncrystallographic symmetry present in the crystal. See Materials and methods for details.

f According to the criterion of MolProbity (59).

### Noncanonical jellyroll folds of IAPV VP1 and VP3 subunits and comparison to virions of cripaviruses.

**FIG 2:**
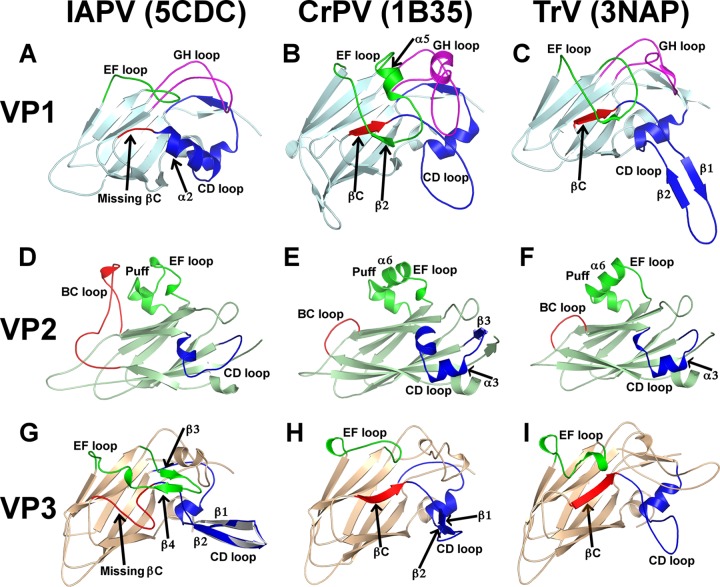
Comparison of capsid proteins of IAPV, CrPV, and TrV. (A to C) VP1 of IAPV contains a loop and α-helix 2, highlighted in red and blue (A), that replace β-strand C in VP1 of CrPV (B) and TrV (C). The EF loop in VP1 of IAPV (A), highlighted in green, is 18 residues shorter than that of CrPV and TrV and lacks α-helix 5 (B and C). The GH loop of IAPV VP1, shown in magenta (A), is 9 and 7 residues shorter than those of CrPV (B) and TrV (C). (D to F) The EF loop, or puff, of IAPV VP2, highlighted in bright green (D), lacks α-helix 6, which is present in CrPV (E) and TrV (F). (D) The BC loop of IAPV VP2 is 15 residues longer than those of CrPV and TrV and interacts with the CD loop. (D and F) The CD loop of IAPV VP2 lacks α-helix 3 and β-strand 3, which are present in CrPV and TrV. (G to I) The VP3 subunit of IAPV lacks β-strand C, which is replaced by a loop, highlighted in red (G); the IAPV VP3 EF loop, shown in green, is 12 residues longer than those in CrPV (H) and TrV (I) and contains two β-strands and a short α-helix. (G) Strands β1 and β2 in the CD loop of IAPV VP3, shown in blue, form the most prominent surface feature of the IAPV virion. The structure of the CD loop from the pentamer crystal form (shown in gray) is superimposed on the virion structure.

**FIG 3:**
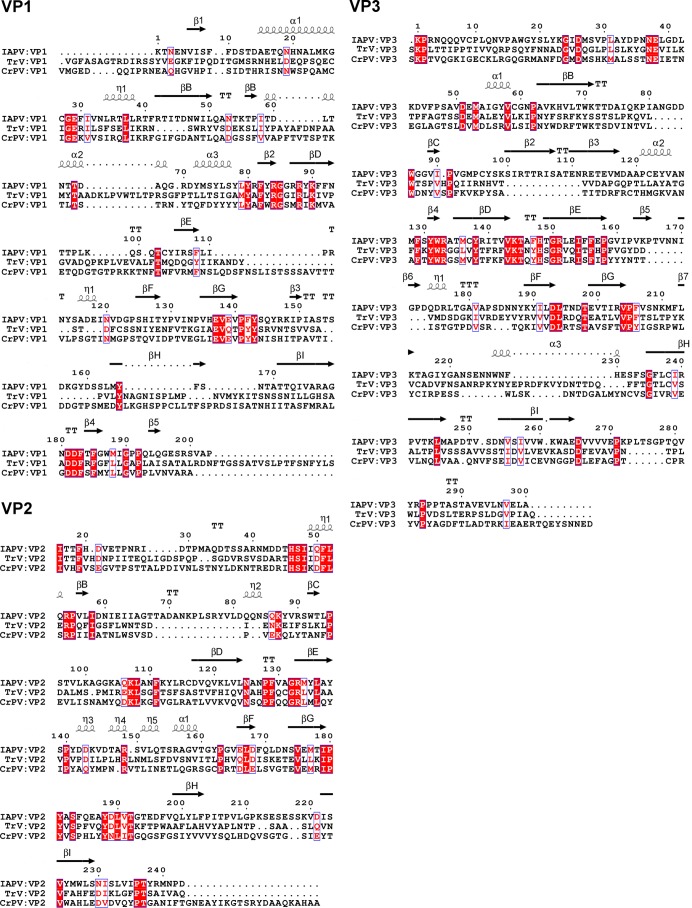
Structure-based alignment of the coat protein sequences of IAPV, CrPV, and TrV. White letters with a red background represent conserved residues, and red letters with a white background represent residues with conserved properties. Secondary-structure elements of IAPV are indicated above the sequence. The arrows represent β-strands, and the spirals represent the α-helices.

While the structures of two dicistroviruses from the genus Cripavirus, CrPV and TrV, have been determined previously (14, 15), IAPV represents the first structurally characterized member of the genus Aparavirus. IAPV shares less than 25% sequence identity with CrPV and TrV (Table 2) and has a different surface topology (Fig. 1A to C). The IAPV virion is spherical, with plateaus around the icosahedral 5-fold and 3-fold axes. There are depressions on the IAPV capsid surface around the icosahedral 2-fold axes similar to those identified previously in TrV (Fig. 1A and C). However, in CrPV, the depressions are partly filled with residues from the C terminus of VP2 (Fig. 1B and E). The most prominent features of the IAPV virion are spikes located between the icosahedral 5-fold and 3-fold axes of symmetry that rise about 20 Å above the virion surface (Fig. 1A). The spikes are formed by two antiparallel β-strands from the CD loop of VP3 and a C-terminal β-strand of VP1 (Fig. 1D and 2A and G). The corresponding β-strands in the CD loop of VP3 in CrPV are 9 residues shorter (Fig. 2H), whereas in TrV, the β-strands are not formed at all (Fig. 2I and 3). The CD loops in IAPV have higher temperature factors in both the virion and pentamer crystal forms (127 and 59 Å^2^, respectively) than the rest of the capsid (120 and 39 Å^2^, respectively), indicating their higher flexibility. As the most prominent features of the IAPV virion, the CD loops might function in receptor binding. The structure of the CD loops is the same in the IAPV virions and the pentamer crystal form (Fig. 2G). The most prominent surface feature formed by IAPV subunit VP2 is the EF loop, which is, according to picornavirus convention, named the puff. The puffs of CrPV and TrV contain short α-helices, α5, α6, and α7 (Fig. 2E and F). However, in IAPV, helix α6 is replaced by a loop (Fig. 2D). The BC loop of IAPV VP2 is 15 residues longer than those in CrPV and TrV and interacts with the CD loop (Fig. 2D to F). Furthermore, the CD loop of IAPV VP2 lacks α-helix 3, which is present in CrPV and TrV VP2 subunits (Fig. 2D to F).

**TABLE 2:** Sequence and structural similarity comparison of capsid proteins of IAPV to those of dicistroviruses and picornaviruses

Virus	Comparison to^*a*^:				
	IAPV	CrPV	TrV	Poliovirus type 1	HRV14
IAPV		2.6/63	1.9/80	2.6/65	2.7/54
CrPV	23		1.8/82	2.6/60	2.6/69
TrV	22	29		2.4/64	2.3/66
Polio virus type 1	11	13	16		1.0/95
HRV14	12	13	14	49	

a Top right: root mean square (RMS) deviations (Å) of superimposed Cα atoms of the respective three-dimensional (3D) structures. The second number indicates the percentage of available amino acid residues used for the calculations. The limit for inclusion was set to 3.8 Å. Bottom left: percent identity between the respective virus coat protein sequences. Gaps were ignored in the calculation. The icosahedral asymmetric units consisting of subunits VP1 to -4 were used in the comparison as rigid bodies. HRV14, human rhinovirus 14.

Unlike capsid proteins with the jellyroll fold of other viruses studied so far (14, 15, 45–47), VP1 and VP3 of IAPV exhibit noncanonical jellyroll folds composed of seven instead of the conventional eight antiparallel β-stands (Fig. 2A and G). The absence of the eighth β-strand in IAPV VP1 and VP3 could be observed both in the 2.7-Å-resolution structure of the pentamer and in the 4.0-Å-resolution structure of the entire virion. β-Strand C in subunit VP1 of IAPV is replaced by a loop and an α-helix that extends into the CD loop (Fig. 2A). The corresponding regions of CrPV and TrV VP1 subunits contain β-strand C (βC), which interacts with a short β-strand, β2, that is part of the EF loop from VP1 (Fig. 2B and C). However, the EF loop of IAPV VP1 is 16 residues shorter than that of CrPV and lacks the β2 strand and α-helix 5 (Fig. 2A and B). The absence of β-strand 2 and its putative stabilizing interaction with residues that form βC in CrPV might enable the corresponding residues in IAPV to adopt a main-chain conformation that does not resemble the β-strand. The EF loop of TrV VP1 is intermediate in size between those of IAPV and CrPV (Fig. 2C). In addition, the GH loop of IAPV VP1 is 9 and 7 residues shorter than the GH loops of CrPV and TrV, respectively (Fig. 2A to C). As a consequence of the relatively short loops of IAPV VP1, it contains only 208 residues, whereas VP1 of CrPV consists of 260 residues and that of TrV has 264 residues (Fig. 3).

β-Strand C in IAPV subunit VP3 is replaced by an elongated loop that forms the capsid surface (Fig. 2G). Moreover, the EF loop of IAPV VP3 is 12 residues longer than those of CrPV and TrV and contains two short β-strands, β3 and β4, that form an antiparallel β-sheet (Fig. 2G). Residues from the EF loop interact with the CD loop of IAPV VP3 (Fig. 2G). In contrast, the residues from the shorter EF loops of CrPV and TrV do not interact with residues from β-strand C (Fig. 2H and I). The unique features affecting the fold of IAPV subunits VP1 and VP3 are exposed at the virion surface and might, therefore, represent functional adaptations to the receptor binding.

**FIG 4:**
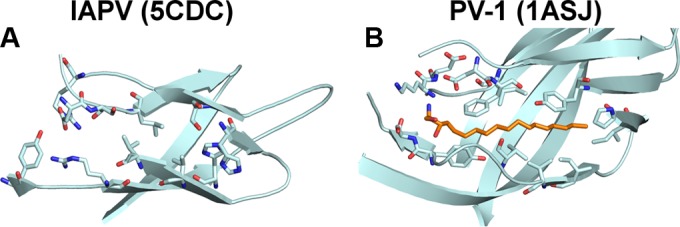
VP1 of IAPV does not contain a hydrophobic pocket. VP1 of IAPV (A) and poliovirus type 1 (B) are shown in cartoon representations. The pocket factor in poliovirus type 1 is shown as a stick model in orange. The residues that form the cores of the subunits and, in the case of poliovirus type 1, interact with the pocket factor are shown as sticks.

Dicistroviruses are structurally and genetically related to vertebrate picornaviruses, for which numerous capsid-binding inhibitors have been developed (48). Compounds that bind into a hydrophobic pocket within VP1 can inhibit receptor binding and/or genome release of some picornaviruses (49–52). However, such a hydrophobic pocket is not formed in IAPV VP1 subunits (Fig. 4). Similarly, the hydrophobic pockets were not observed in VP1 subunits of CrPV and TrV (14, 15). This suggests that capsid-binding inhibitors may not be effective as antivirals against honeybee viruses from the genus Aparavirus.

### Maturation cleavage of VP0 into VP4 and VP3.

**FIG 5:**
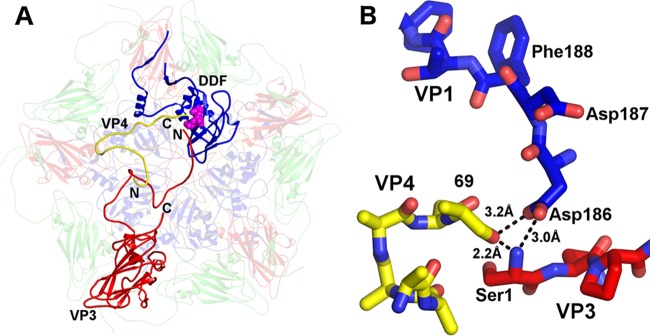
Residues Asp-Asp-Phe of VP1, constituting the putative proteolytic site that might mediate the cleavage of VP0 into VP3 and VP4, are positioned close to the N terminus of VP3 and the C terminus of VP4 from another protomer related by an icosahedral 5-fold axis of symmetry. (A) Cartoon representation of a pentamer of capsid protein protomers viewed from inside the virion. (B) Detail of the putative active site in stick representation. VP1 subunits are shown in blue, VP2 in green, VP3 in red, and VP4 in yellow.

The maturation of the capsids of many viruses from the order Picornavirales is dependent on cleavage of capsid protein VP4 from the N terminus of a precursor subunit, called VP0. In picornaviruses, the VP0 cleavage generates the proteins VP4 and VP2, while in dicistroviruses, the precursor cleavage generates VP4 and VP3 (14, 15). It has been proposed previously that a conserved Asp-Asp-Phe (DDF) motif, which is part of the VP1 subunit that is exposed to the virion cavity, is involved in the VP0 cleavage (14, 15, 17). The cripaviruses CrPV and TrV contain the DDF sequence in a loop immediately following β-strand I of VP1. TrV has an additional, second DDF sequence in a loop following β-strand I of VP3 (14, 15). IAPV has the DDF sequence in VP1, formed by residues 186 to 188, located in a position similar to those in the DDF sequences of TrV and CrPV. Asp186 of the IAPV DDF motif is located close to the N terminus of VP3 and the C terminus of VP4 from the neighboring protomer, indicating that it may catalyze the cleavage (Fig. 5). The conformation of the DDF site is similar to that observed in flockhouse virus, even though it has completely different capsid morphology, in which the Asp residue performs an autocatalytic cleavage that is necessary for capsid maturation (53). The relative positioning of the DDF motif in IAPV and the VP4 C terminus and VP3 N terminus indicates that the formation of pentamers is sufficient to achieve the optimal spatial arrangement of the catalytic center and substrate for the cleavage (Fig. 5A). However, the mechanism that ensures that the VP0 cleavage occurs only in virions containing the RNA genome (14, 53) remains to be determined.

### Putative roles of VP4 and the N terminus of VP1 in delivery of the IAPV genome across the biological membrane.

**FIG 6:**
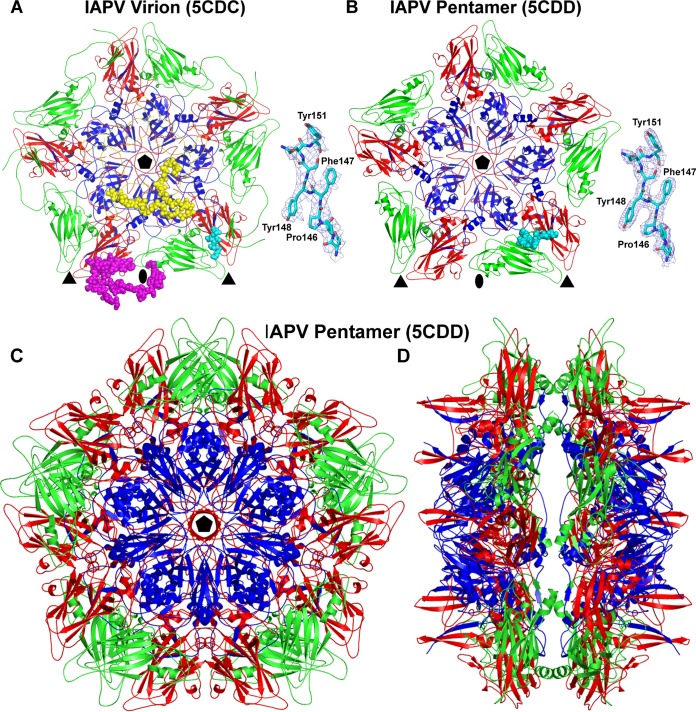
(A and B) Comparison of pentamer structures from the IAPV virion (A) and separately crystallized pentamers of capsid protein protomers (B). VP1 subunits are shown in blue, VP2 in green, VP3 in red, and VP4 in yellow. The differences between the two structures are highlighted in one of the protomers: nine N-terminal residues of VP1 are shown as space-filling spheres in cyan and 64 N-terminal residues of VP2 as space-filling spheres in magenta. (B) VP4, shown in yellow, is missing from the pentamer structure. (A) The N-terminal arms of VP2 subunits mediate interpentamer interactions in the virion. The positions of 5-fold, 3-fold, and 2-fold icosahedral symmetry axes are indicated by pentagons, triangles, and ovals, respectively. The insets show representative electron densities and corresponding models in stick display. (C and D) Top (C) and side (D) views of the decamer assembly of capsid protein protomers from the pentamer crystal form.

**FIG 7:**
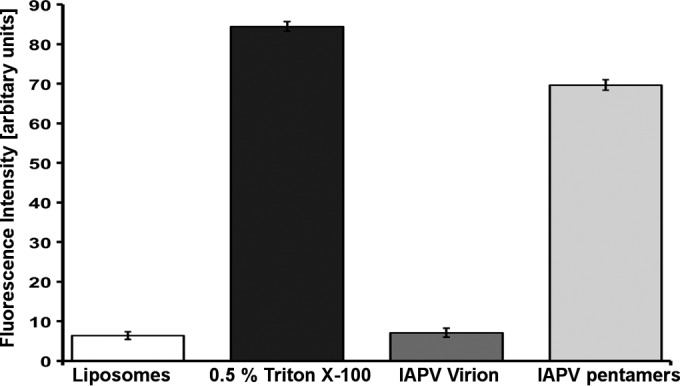
Heat-disrupted IAPV virions induce permeabilization of liposomes. Liposomes filled with self-quenching fluorescent dye were incubated with the detergent Triton X-100, native IAPV virions, and IAPV virions heated to 62°C for 5 min. Permeabilization of the liposomes was detected as an increase in fluorescence. See Materials and Methods for details.

The delivery of dicistrovirus genomes into the host cell cytoplasm has not been studied. However, findings from related mammalian picornaviruses showed that VP4 subunits are released together with the genome and that the N termini of VP1 are externalized on the capsid surface at the beginning of the infection. The genome release of many picornaviruses results in the formation of empty capsids, the so-called B particles (54). However, the empty capsids of some picornaviruses and dicistroviruses disassemble into pentamers of capsid protein protomers (55, 56). Furthermore, pentamers of capsid protomers were also shown to be capsid precursors (57, 58). Even though the crystallization of native IAPV virions was attempted, one type of crystal was formed from pentamers of capsid protein protomers (Table 1). Dimers of pentamers of capsid protein protomers very similar to the crystallized form of IAPV capsid proteins (Fig. 6) were previously observed as disassembly products of TrV (56). The two pentamers are held together by interactions of residue His61 of VP2 with Val11 from the N terminus of VP1 and of Gln65 from VP2 with Asp198 from VP3 (Fig. 6D). It is of particular interest that capsid protein VP4 is missing from the pentamers and that 9 residues from the N-terminal arm of the VP1 subunit have a different structure than in the virion (Fig. 6A and B). It is therefore possible that the extended exposure of IAPV to the crystallization conditions of 0.1 M sodium acetate at pH 4.5 induced the virions to release their genomes and disassemble into pentamers in a process mimicking natural genome release. The detachment of VP4 from the pentamers and changes in the structure of the VP1 N termini indicate that these peptides in IAPV might have functions similar to those in picornaviruses (26, 32, 33). This speculation is reinforced by the observation that, whereas native IAPV virions do not affect the integrity of liposomes *in vitro*, heat-dissociated IAPV particles induce liposome disruption (Fig. 7).

VP2 of IAPV has an elongated N-terminal arm that mediates contacts between the pentamers of the capsid protein protomers (Fig. 6A). The N-terminal arm of the VP2 subunit reaches around an icosahedral 2-fold axis into a neighboring pentamer; approaches a 3-fold axis; and forms two β-strands, β1 and β2, that extend the β-sheet HEF of a VP3 subunit from the same pentamer that contains the VP2 subunit (Fig. 6A). The electron density corresponding to residues 1 to 58 from the N terminus of VP2 is not resolved in the pentamer crystal form (Fig. 6B). This verifies that interpentamer contacts are required to maintain the structure of the VP2 N terminus and its stabilizing function within the virion.
